# W. H. Van Buren, M.D., LL.D.

**Published:** 1883-04

**Authors:** 


### Obituary.

William Holme Van Buren, M.D., LL.D., New York.—Professor William H. Van Buren, M.D., LL.D. (Yalen), died in this city on the morning of March 25, 1883, within twelve days of the completion of his sixty-fourth year.

Born April 5, 1819, of a line of medical ancestors, Dr. Van Buren reached a position in the profession of the highest distinction.

His graciousness of manner, his dignified dexterity as an operator, his wide command of language as a lecturer, his elegance of diction as an author, the magic in the sick-room, will be long and pleasantly remembered by those who came in contact with him.

He entered Yale with the class of '38, but did not graduate; yet the college, in recognition of his literary attainments and distinguished position, decorated him with the honorary degree A.M. in 1866, and later, in 1878, conferred upon him the highest title in her gift, LL.D.

The University of Pennsylvania, in 1840, graduated him in medicine after his return from a tour in the Paris hospitals. He then served in the army until 1845, in which year he came to New York to assist the late Valentine Mott in the work of his clinique in the medical department of the University of New York.

During thirty-eight years he labored in the rich surgical fields furnished by this great metropolis, reaching distinguished honors which accumulated upon him as he advanced in life.

Among these may be mentioned the professorship of anatomy in the University, and of the science and art of surgery as well as of genito-urinary surgery and syphilis in the Bellevue Hospital Medical College. He did creditable work as an active surgeon to the Bellevue, the New York and St. Vincent's Hospitals, and has been consulting surgeon to St. Vincent's, New York, Bellevue, Charity, the State Woman's and the Presbyterian Hospitals. He was for many years active in medical society work, being Vice-President of the New York Academy of Medicine and President of the Pathological Society. He was a corresponding member of the Society of Surgery of Paris.

His first literary efforts were translations of French works, but soon able essays upon matters of personal experience appeared from his pen in the various medical magazines—essays marked by the honesty of the observations reported as well as their graceful elegance of expression. His "Contributions to Practical Surgery" appeared in 1865, his "Lectures on Diseases of the Rectum" in 1870, and again, in much better form, as a second edition in 1882. In 1874 he appeared as joint author with his junior partner in a text-book on "Genito-Urinary Surgery," and finally, his latest and perhaps ablest production on "Inflammation" came out in "Ashhurst's Encyclopædia of Surgery" only a short time before his death.

The credit of being the author of the tunneling of urethral instruments attaches to his name. This improvement is perhaps second to none in modern urethral surgery.

He leaves a wife—a daughter of the late Valentine Mott—and two married daughters to deplore his loss.

His death was due, indirectly, to apoplexy, which occurred in the spring of 1882, directly, to degenerative changes about the injured cerebral focus, changes which set in with the beginning of the present year. He appreciated the approach of death, and wished for it.

